# Open Reduction and Internal Fixation of Intraarticular Fractures of the Humerus: Evaluation of 33 Cases

**DOI:** 10.5812/traumamon.5278

**Published:** 2013-01-15

**Authors:** Keykhosro Mardanpour, Mahtab Rahbar

**Affiliations:** 1 Department of Orthopedics, Kermanshah University of Medical Sciences, Kermanshah, IR Iran; 2 Department of Pathology, Kermanshah University of Medical Sciences, Kermanshah, IR Iran

**Keywords:** Elbow Joint, Fracture Fixation, Internal, Osteotomy

## Abstract

**Background:**

Standard treatment of type C elbow fractures is open reduction and internal fixation using reconstruction plates and pins.

**Objectives:**

The aim of this study was to evaluate the functional outcome following internal fixation of intraarticular fractures of the distal humerus (AO Type C) with a minimum follow-up of three years. A retrospective evaluation was undertaken.

**Patients and Methods:**

Thirty-three patients (28 males, 5 females; mean age 34.3years) type C elbow fractures were treated and observed over a period of three years. Six fractures were open and 27 closed; causes were falls (7 cases), traffic accidents (22 cases) and altercation (4 cases). All operations were performed using a posterior approach with an olecranon osteotomy. Mean duration of follow-up was 18 months (range 6–36). Mean duration of fracture healing was 2.3 months (range 2–4). Functional outcomes were assessed by Jupiter criteria.

**Results:**

Excellent results were found in 69.7% (23 cases), very good reaults and good results were found in the remaining 30.3% (10 cases). Three of 33 patients 9% (3 cases) presented postoperative complications. No patient exhibited symptoms of ulnar nerve injury following surgery. One patient had cubitusvarus deformities and one case had heterotopic ossification. One patient had malunion and one case had deep infection.

**Conclusions:**

Complications were minimal and outcomes were satisfactory in patients with type C distal humerus fractures who underwent bilateral plate fixation via a posterior approach.

## 1. Background

Distal humerus fractures have been estimated to be 287 per 100,000 person/year in the United States (1).Although the incidence is very low, this type of fracture is often associated with neurovascular injuries (2, 3). Furthermore, most manual methods of reduction are unsatisfactory for type C distal humerus fractures; hence, surgical treatment is preferred (4).Previous treatment methods of closed reduction with immobilization, traction and limited internal fixation have caused significant functional impairment with loss of range of movement (5-7).

Restoration of normal anatomy and early movement may lead to a better functional outcome of distal humerus fractures. Different methods of internal fixation and open reduction have been reported using Kirschner wires, screw fixation and single plates (8-10). The improved techniques for internal fixation introduced by AO/ASIF helped surgeons to perform early mobilization and obtain predictable functional results. Two column plates at 90° to one another in complicated elbow fracture have become standard treatment (11).

Open anatomical reduction followed by internal fixation with a reconstruction plate is the most common approach (12, 13). Distraction reduction with external fixation can be employed for patients with serious fracture displacement; in those who have severe soft tissue swelling, internal fixation should be delayed until swelling subsides (14). Operation may have to be delayed several days or one to two weeks.

There are two significant factors which influence prognosis. The first one is delay in surgical repair following injury and the second is difficulty in obtaining adequate surgical exposure. Therefore proper surgical approach and timing are important factors for obtaining good results.

## 2. Objectives

There are several surgical approaches to repair type C fractures of the distal humerus. One of these is a posterior approach with transolecranon osteotomy (15, 16). In this study, we treated type C distal humerus fractures using a posterior approach and bilateral plate fixation and assess the outcomes.

## 3. Patients and Methods

From March 2009 to May 2012, thirty-three patients with type C distal humerus fractures were treated at our department. All underwent bilateral plate fixation. All patients with type C open fractures of the elbow were treated except those with other life-threatening injuries like cerebral trauma, visceral injuries of the chest, abdomen or other sites. Life threatening injuries were treated first and then fractures were repaired when patient’s were stable. In patients with closed fractures, the operation was performed after swelling subsided.

The anaesthetized patient was placed in a supine position. The upper limb was placed in front of the chest, with shoulder and elbow in flexion. A midline posterior skin incision was made beginning 6 cm proximal to the olecranon, extending distally, skirting the ulnar aspect of the tip of the olecranon, and continuing for a further 6 cm along the subcutaneous border of the ulna. The ulnar nerve was identified and carefully dissected from the cubical tunnel. Dissection was performed along the tricepsbrachii muscle bilaterally to the proximal ulna; and osteotomy was performed 3.0 cm distal to the tip of the olecranon. The proximal part of the olecranon and its attached triceps tendon were retracted proximally to expose the distal humerus. The distal humerus and elbow were exposed entirely, the intercondylar fracture was first reduced and temporarily fixed by using K-wire to restore the smoothness of the articular surface and convert the type C fracture to a type A fracture. The type A fracture was reduced and fixed with bilateral plates to ensure the stability of the medial and lateral columns of the distal humerus.

Bilateral plates were pre-bent according to the morphology of the distal humerus. Medial and lateral plates were placed on the medial and posterolateral sides of the humerus at 45 to 90º angle to one another. At the end of the procedure, reconstruction of the soft tissues was performed. The olecranon was then reduced and fixed by K-wire and tension band wire. The medial portion of the triceps was brought back to the olecranon and the ulnar nerve was seen to fall into its anatomical position. Reattachment of the triceps to the olecranon allowed adjustment of soft-tissue tension. In the case of open fractures, antibiotics were administered for five to seven days. After the operation, the patient’s elbow may be splinted or casted for a short period of time. Patients may wear a sling if it provides them more comfort. Pain medications may be provided. Stitches or staples were typically removed 10 to 14 days after the operation, but this depends on the preference of the surgeon. Active exercises for the elbow and forearm usually began shortly after the operation; sometimes as early as the next day or 48 hours. It is extremely important that once exercise is started, it must be done multiple times every day. Sometimes, the patients visited a physical therapist. The exercises only make a difference if they are done regularly. Patients were usually restricted from lifting objects with the injured arm for 6 to 12 weeks.

Two weeks after the operation, follow-up took place every 4 weeks until fracture healing occurred and thereafter every 6 weeks. Implanted plates, K-wires and tension band wiring were removed 15 to 18 months after fracture healing. Final follow-up was performed approximately 1 year later. These patients were assessed retrospectively by clinical evaluation, exploration of x-rays based on the AO classification and functional outcome based on Jupiter criteria (Table 1)(17).

**Table 1:** Functional Scoring System, Jupiter Criteria

	Excellent	Good	Fair	Poor
**Range of Motion(degree)**				
Loss of extension	< 15	< 30	< 40	< 40
Loss of flexion	> 120	> 130	> 90	> 90
**Pain**	None	Slight	Mild activity	Variable
**Disability**	None	Mild	Moderate	Sever

Fracture healing was assessed via local tenderness, pain, abnormal movement, continuous callus formation on control X-ray and by ability to lift and hold a 1 kg object for one minute without deformation at the fracture site. After fracture healing, patients were followed-up every 6 weeks.

## 4. Results

There were 28 male and 5 female patients. The mean age was 43.3 (SD ± 15.9) years, ranging from 16 to 75 years. Six fractures were open and 27 closed. Fracture etiology included falls (7 cases), traffic accidents (22 cases) and altercations (4 cases). According to AO/ASIF classification, all cases were classified as C type fractures. Twelve cases were associated with other fractures, including 5 ulnar shaft fractures, 2 distal radial fractures, 2 fractures of the surgical neck of the humerus, one lumbar vertebral body fracture, and 2 clavicle fractures. Ulnar nerve injury was evident in two patients before operation.

**Figure 1:**
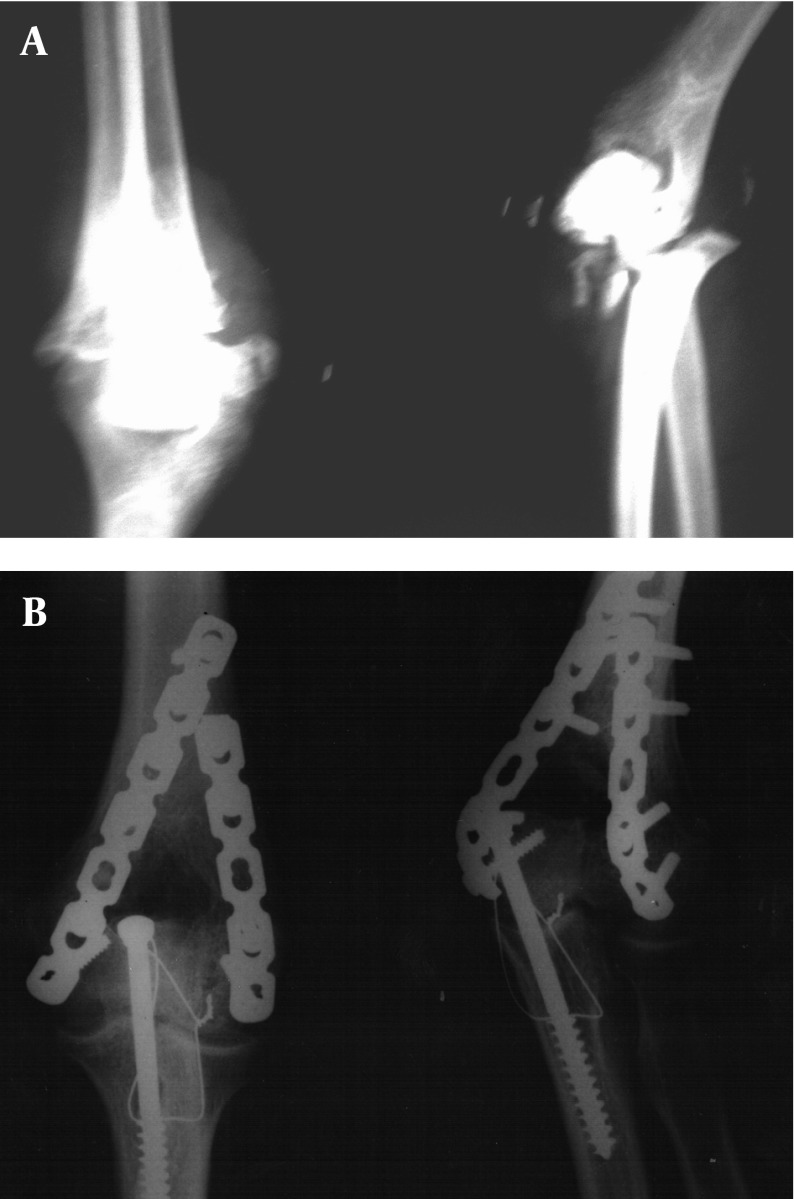
Radiographs Illustrating a Representative Type C Distal Humerus Fracture. Anteroposterior and Lateral Views Following Bilateral Internal Plate Fixation via an Olecranon Osteotomy Approach

The mean duration between injury and operation was about 5 days, ranging from 0 to 7 days. Among 6 patients with open fractures, only 1 case underwent emergency surgery. In this patient, emergency debridement and fracture fixation was performed. Operation was delayed in the remainder until other life-threatening injuries were treated. All operations were performed successfully with no intraoperative complications. Two reconstruction plates were used in all 33 cases (Figure 1).

**Figure 2:**
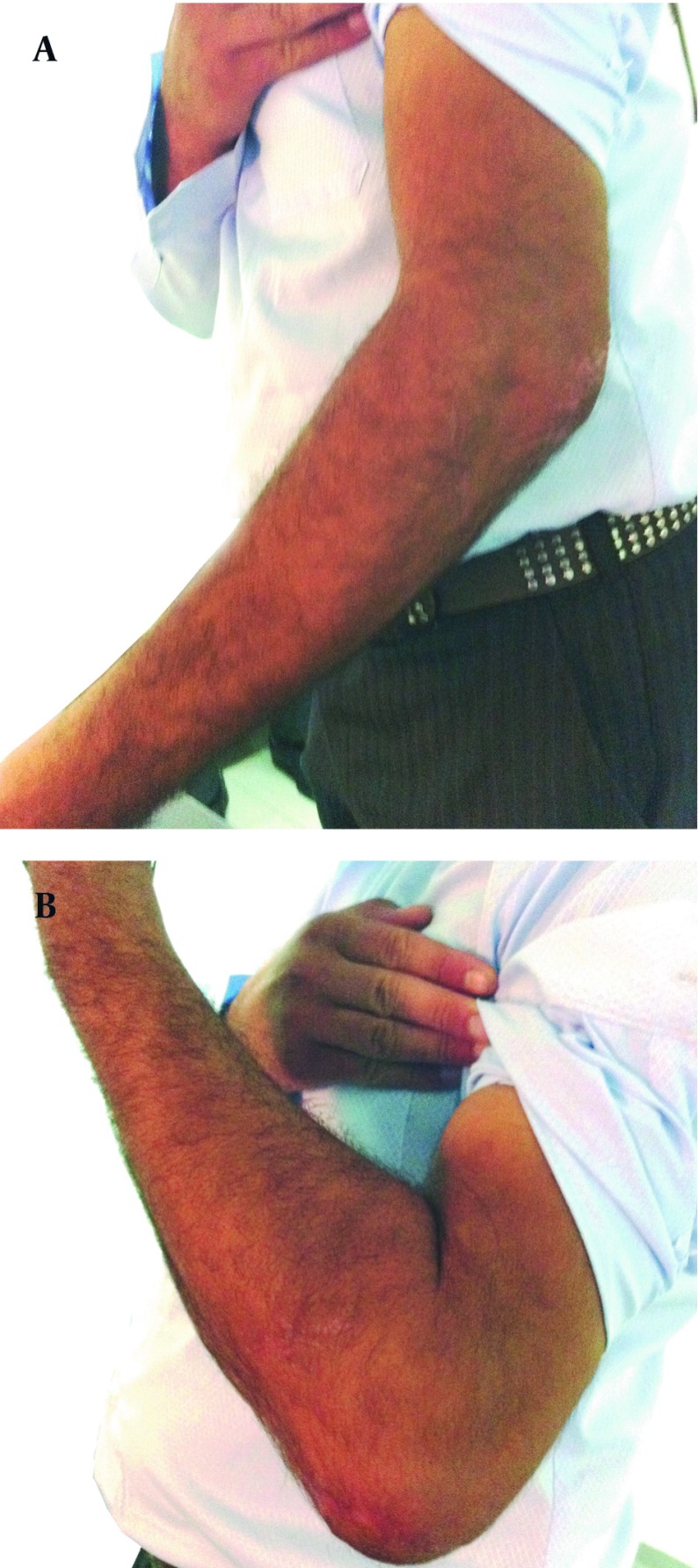
Full Extension and Full Flexion of Treated Elbow Fracture

Excellent results were found in 69.7% (23 cases), very good and good results were found in the remaining 30.3% (10 cases). The mean hospitalization duration was 4 days. Polytrauma was seen in 36.6% (12 cases) of the patients (Figure 2). The mean duration of follow-up was 18 months, ranging from 6 to 36 months. The mean duration of fracture healing was 2.3 months, ranging from 2 to 4 months. Thirty of 33 patients (90.9%) had no postoperative complication. Four of 6 patients with open fractures had other injuries (4 patients with other fractures and 2 of these had ulnar nerve injury). Only 8 of 27 patients with closed fractures had other fractures, while no patient had ulnar nerve injury. After the operation, 1 mild cubitus varus deformity due to heterotopic ossification, 1 superficial infection and 1 case with malunion occurred.

## 5. Discussion

One of the most problematic fractures is intraarticular distal humerus fracture. Many different surgical techniques have been advocated but none of them are optimal. Insufficient stabilization and prolonged immobilization are the main causes of unsatisfactory results. Rigid fixation and early rehabilitation are the most important goals in treatment of type C elbow fracture. In our experience, emergency surgery was sought for each patient with open fracture after debridement. In patients with closed fracture and severe local swelling, olecranon traction was performed first and internal fixation was performed about one week after swelling subsided.

Different approaches have been described for type C distal humerus fracture repair (18, 19). The posterior approach has been used by many surgeons because it exposes the articular surface of the distal humerus sufficiently (20, 21). In our study, posterior approach was used in 33 cases of type C distal humerus fracture. The advantages of this approach are:

1- Protection of the ulnar nerve by the medial part of triceps reduces the possibility of damage to its blood supply.

2- Availability of the two segments of the triceps for the repair allows satisfactory balancing of the medial and lateral sides of the elbow.

3- Reduced risk of postoperative dislocation.

4- Good fracture reduction.

5- The implementation of early functional exercises is possible.

Articular restoration is the most essential step followed by stabilization of the largest columnar fragment. There are several options for fixation between the condyle and humeral metaphysis. These include the use of Y-shaped plates, single plates, double K-wire, and K-wire together with tension band wiring (14, 22).The aim is to facilitate biomechanical reconstruction of the aforementioned two-column structure. Bilateral plate fixation was carried out in all 33 cases in our study. In each case, fracture reduction was satisfactory, fixation was strong and durable, fracture site stable and early post-surgical functional exercise was possible.

There are many complications which have been reported following surgical repair of type C distal humerus fractures. These include infection, nerve injury, joint stiffness, heterotopic ossification and delayed union or nonunion of the ulnar olecranon (23, 24). Kundel et al. reported that the incidences of heterotopic ossification and nerve injury were 49% and 33%, respectively, following open reduction and internal fixation (23). In our study, no patient was found to have symptoms of ulnar nerve injury after the operation. This is well below the rate reported by Kundel et al. (23). This may be due to intraoperative protection of ulnar nerve. The incidence of heterotopic ossification in our study was also lower than that previously reported. Gofton et al. found that 13% of patients with type C distal humerus fractures exhibited postoperative heterotopic ossification (24). The lower incidence in our study may relate to complete intraoperative hemostasis, unobstructed postoperative drainage, and early postoperative functional exercise. In contrast to the findings of distal humeral nonunion in several previous reports (25, 26), no instances of fixation failure were detected in this study. Presumably this was a reflection of strong bilateral plate fixation and satisfactory fracture reduction. One malunion at plate replacement site was apparent. This may have been due to the early post-operative implementation of exercises. Healing ensued in all of these patients following decrease in the level of exercise intensity.

In summary, we found that use of a posterior approach and bilateral internal plate fixation was efficacious for the treatment of type C distal humerus fractures. Early mobilization was possible in the majority of cases, which may be a prerequisite for satisfying functional results. Complications were minimal and healing satisfactory. We advocate the use of this approach for repair of type C distal humerus fractures.
